# Derailed Intraneuronal Signalling Drives Pathogenesis in Sporadic and Familial Alzheimer's Disease

**DOI:** 10.1155/2014/167024

**Published:** 2014-08-27

**Authors:** Tom Van Dooren, Katrien Princen, Koen De Witte, Gerard Griffioen

**Affiliations:** reMYND, Gaston Geenslaan 1, 3001 Leuven, Belgium

## Abstract

Although a wide variety of genetic and nongenetic Alzheimer's disease (AD) risk factors have been identified, their role in onset and/or progression of neuronal degeneration remains elusive. Systematic analysis of AD risk factors revealed that perturbations of intraneuronal signalling pathways comprise a common mechanistic denominator in both familial and sporadic AD and that such alterations lead to increases in A*β* oligomers (A*β*o) formation and phosphorylation of TAU. Conversely, A*β*o and TAU impact intracellular signalling directly. This feature entails binding of A*β*o to membrane receptors, whereas TAU functionally interacts with downstream transducers. Accordingly, we postulate a positive feedback mechanism in which AD risk factors or genes trigger perturbations of intraneuronal signalling leading to enhanced A*β*o formation and TAU phosphorylation which in turn further derange signalling. Ultimately intraneuronal signalling becomes deregulated to the extent that neuronal function and survival cannot be sustained, whereas the resulting elevated levels of amyloidogenic A*β*o and phosphorylated TAU species self-polymerizes into the AD plaques and tangles, respectively.

## 1. Introduction

Alzheimer's disease involves a gradual decline of synaptic function which is clinically presented as dementia [1, 2]. AD brains are defined by the presence of two different protein aggregates: plaques and tangles. Plaques are assemblies of extracellularly deposited A*β* peptides predominantly comprising the A*β*40 and the highly amyloidogenic A*β*42 peptides. These peptides are the products of sequential processing of APP (amyloid precursor protein) by BACE1 and *γ*-secretase [3]. It is generally assumed that in AD homeostasis of A*β*40 and 42 species is altered resulting in increased formation of oligomeric A*β* (A*β*o) and subsequent aggregation into plaques. Tangles comprise intracellular assemblies of hyperphosphorylated TAU, a protein which as monomer—among other functions—binds to and stabilizes microtubules [4, 5]. There is high degree of consensus that in AD kinase and/or phosphatase activities are deregulated, resulting in hyperphosphorylation of TAU. TAU then loses its ability to bind to microtubules and consequently acquires a high propensity to oligomerise and further aggregate in tangles [6].

Decades of AD research have culminated in a wealth of data on virtually every aspect of AD etiology and pathogenesis. This has led to detailed insights into the mechanisms of AD, such as APP processing or TAU-phosphorylation, but a coherent picture encompassing AD pathology (i.e., cause/etiology, mechanisms of development, structural changes of neurons, and clinical manifestations) is still in its infancy. This review attempts to contribute to this discussion by proposing mechanisms that may help to design a conceptual framework of AD pathology.

## 2. Intraneuronal Signaling and Endocytosis Are Dysregulated in AD Leading to Increased A***β***o Formation and TAU-Phosphorylation, which in Turn Further Derange Signalling

### 2.1. A*β* Oligomers Impact Intraneuronal Signalling in Familial AD

Sporadic AD is often phrased as idiopathic to emphasise that the cause of the neuronal degeneration and symptoms is unknown. Although undoubtedly true for individual patients, epidemiological studies have revealed several positive and negative AD risk factors which may hold clues as to the mechanism of AD pathogenesis (Table 1). Remarkably, these risk factors are highly diverse, consisting of genetic, lifestyle, and environmental cues with various degrees of disease penetrance. For instance, ApoE variants and zygosity are either protective against or strongly increase the risk of AD [7]. Ageing, smoking, traumatic brain injury, or metabolic diseases such as diabetes are examples of nongenetic modifiers [8]. In rare familial cases mutations in APP or its processing machinery comprise highly penetrant risk factors which in itself suffices to trigger AD.

Irrespective of their nature and origin, at a certain point these risk factors converge to a common mechanism involving synaptic failure, A*β* and TAU pathology, and subsequent neuronal loss. Thus, a key question of understanding AD is not what causes AD, as these are multifactorial and heterogeneous among patients, but how these may converge mechanistically to trigger AD pathology. Once understood, principally every condition impacting this mechanism could be considered as contributing to AD and effective therapeutic options targeting this mechanism could be rationalised for treating AD.

**Figure 1:**
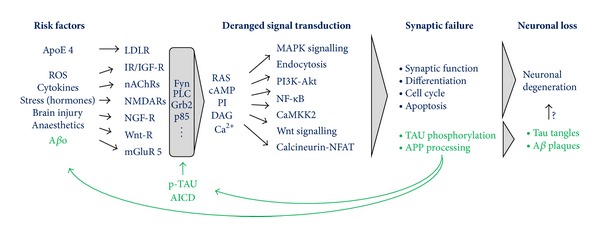
APP, its processing products and TAU are part of an intraneuronal signalling network required for neurogenesis, neuronal function, and survival which go awry in AD. A*β*o and AD risk factors modulate receptor mediated intraneuronal signalling and endocytosis which impacts A*β* homeostasis and TAU-phosphorylation. TAU-hyperphosphorylation leads to decreased microtubule binding, somatodendritic redistribution, and altered signalling. Apart from a modulatory role of A*β*o, AICD, and phosphorylated TAU on signalling, their formation is also controlled by signalling implying a positive feedback loop which could overtime lead to a dysfunction of signalling cascades underlying synaptic integrity and neuronal survival. High levels of A*β*o and hyperphosphorylated-TAU species will, due to their intrinsic amyloidogenic propensity, ultimately aggregate into plaques and tangles. Risk factors which impact these signalling processes, either directly or indirectly (i.e., through impacting A*β*o levels), will set off this cascade of events culminating in synaptotoxicity and pathology. Note that the schematic is highly simplified and intended to depict general principles. For a more exhaustive insight into the signalling pathways impacted in AD, see [30]. Abbreviations are as follows: LDLR: low density lipoprotein receptor; IR: insulin receptor; IGF-R: insulin-like growth factor receptor; nACHR: nicotinic acetylcholine receptor; NMDAR: N-methyl-D-aspartate receptor; NGF-R: nerve growth factor receptor; Wnt: Wingless Int; PrPc: cellular prion protein; RAS: rat sarcoma; cAMP: cyclic adenosine monophosphate; PI: phosphoinositides; DAG: 1,2-diacylglycerol; mGluR5: metabotropic glutamate receptor; MAPK: mitogen-activated protein kinase; PI3K: phosphoinositide 3-kinase; NF-*κ*B: nuclear factor kappa-light-chain-enhancer of activated B cells; CamKK2: calcium/calmodulin-dependent protein kinase 2; and NFAT: nuclear factor of activated T-cells.

The discovery of genetic risk factors causing early onset AD has been extremely instructive to reveal such common mechanism since in these exceptional cases only one defined cause, namely, altered APP processing, triggers AD providing an relatively “simple” paradigm to investigate pathogenesis. From numerous studies on the mechanism of APP-dependent neurotoxicity, a picture emerges in which A*β*o, but not plaques or monomers, comprises prime candidates responsible for synaptic failure, TAU-phosphorylation, and neuronal loss [9–15]. Also the AICD, another APP processing product, may play a role here [16–18]. A*β*o has been shown to bind directly to, or modulate indirectly, numerous neuronal receptors [19] implying that these impact synaptic signalling cascades including MAPK, Akt, Wnt, and Rho pathways (summarized in Table 2 with references and Figure 1). It appears that A*β*o acts as a nonspecific pathological receptor ligand/agonist, both at the pre- and postsynaptic membrane. In addition, A*β*o binds to membranes directly which appear to involve GM1 ganglioside and as such are thought to induce structural and functional changes which may impact Ca^2+^ signalling and synaptic plasticity [20, 21]. A global impact of A*β*o on different signalling pathways and their respective signalling components is consistent with the widely held view that kinase and phosphatase activities are imbalanced early on in the pathogenesis in diseased neurons [22], resulting in improper hyperphosphorylation of downstream substrates including TAU [6, 23].

Also APP processing itself is controlled by signal transduction pathways. GPCR's, like GPR3 and *β*2-adrenergic, receptors mediate their effects on APP processing through interaction with *β*-arrestin and *γ*-secretase [24, 25]. Activation of JNK3 MAPK by A*β*o phosphorylates APP at T668, thereby increasing its endocytosis and subsequent processing [26, 27]. Also, Ras-Erk1/2 and PI3K-Akt signalling pathways activate APP-expression [28] or PS1, a subunit of the APP processing machinery [29]. These results suggest that A*β*o increases its own formation by modulating APP processing through these signalling pathways (Figure 1).

### 2.2. Signalling and Endocytosis: Intimate Partners in Crime

Cell signalling and endocytosis are increasingly recognized as intertwined and bidirectionally controlled processes [31–33]. Receptor internalisation by endocytosis is a common response upon ligand binding to desensitize cells. Internalised receptors are shuttled to early endosomes, which act as a sorting station for recycling to the plasma membrane or to the lysosome for degradation. Signal propagation is not restricted to the plasma membrane but (may) continue(s) after internalisation. Endosomes marked with active signalling pathways, referred to as signalling endosomes [34], prolong and even intensify signalling while transported through the cell. To illustrate this, activation of the NGF receptor at the presynaptic membrane transiently activates RAS-Erk1/2 signalling [31, 35]. Upon internalization, signalling is sustained as NGF-receptors remain actively coupled with Erk1/2, however, in this context via RAP1, while being transported to the nucleus in order to phosphorylate substrates such as CREB and Erk5. Another example of a close link between signalling and endocytosis entails Wnt signalling. Here, internalization of activated receptors is required to control GSK3 activity and *β*-catenin stability [36, 37]. Conversely, signalling controls the endocytic pathway itself by impacting the phospholipid turnover. Increases of PIP3 by activation of PI3K allows (apart from Akt) membrane recruitment of Rho and Arf GEFs and GAPs in turn modulating their respective GTPases involved in vesicular trafficking (among other functions such as cytoskeletal rearrangement) [38].

As discussed above, intracellular signalling is deregulated in AD by risk factors and A*β*o and thus inevitably will impact endocytosis. Indeed, sporadic AD is characterised by an abnormal activation of the endocytic pathway, with associated increases in PI3K and RAS signalling and Rab5 levels, and comprises early neuropathological alteration even before A*β* pathology ensues [39–41]. Consistent with its role as a pathological receptor ligand, A*β*o also increases internalisation of receptors by endocytosis [42–44]. An even more general effect on the endocytic pathway is expected by A*β*o triggered receptor-mediated activation of phospholipid signalling through neurotrophin or insulin signalling pathways [45–48]. Likewise, stress-activated p38 MAPK, a kinase activated in AD, stimulates Rab5 which leads to acceleration of endocytosis [49].

Note that AICD, another APP processing product, activates signalling through interaction with Shc and Grb2, adaptor proteins that link with Ras/Erk1/2 and PI3K/Akt pathways (reviewed in [18]) and (perhaps as a consequence) trigger endocytic dysfunction [16]. In addition, GSK3 is also increased by AICD through inhibition of Wnt signalling [50]. Thus, apart from A*β*o, other APP processing products also impact signalling and endocytosis in AD and may therefore, at least in part, contribute to the development of AD pathology independently of A*β* [17].

As BACE1 and *γ*-secretase are localised at endosomes, amyloidogenic, neurotoxic processing of APP requires endocytosis [51–54] and is controlled by vesicular trafficking [55]. Conditions that alter the residence time and/or levels of APP or its processing enzymes at the endocytic compartment impact A*β* production or clearance accordingly [55–58]. For instance, Arf6, a small GTPase controlled by phospholipid signalling, mediates endosomal sorting of BACE1 and thereby APP processing [59]. Or the already abovementioned JNK-driven phosphorylation of APP at T668 facilitates its endocytosis and processing [27]. Similarly, ApoE receptors facilitate the internalization of APP to the endosomal compartment [60].

Taken together, the processing of APP is controlled by signalling pathways which impact expression and endocytic localisation of APP and its processing machinery. Thus, aberrant activation of pathways that increase endocytic APP levels also allows more processing and hence elevates A*β*o and AICD formation. This model implies a positive feedback loop as APP processing itself is activated by its own products through signalling (Figure 1). In this way, subtle genetic or nongenetic AD risk factors which lead to relatively small perturbations of signal transduction pathways could, if unchecked, trigger over time a large buildup of A*β*o/AICD and thus amplify these subtle alterations into large derangements of signalling and associated endocytosis.

### 2.3. Derailed Intraneuronal Signalling Is a Common Denominator in Sporadic and Familial AD

As discussed above, in familial AD increased formation of A*β*o impacts receptor-mediated signalling. In addition, APP, its processing machinery, and the AICD impact signalling independent of A*β* formation (reviewed in [61]). For instance, PS1, a subunit of the *γ*-secretase complex, cleaves numerous transmembrane signalling receptors and transducers other than APP CTFs, including Notch, cadherins, ErbB4, LDL receptor related proteins, and so forth [62, 63]. In addition, PS1 and PS2 impact signalling pathways directly. Deletion of PS1 and/or PS2 activates Erk1/2 activity in cell line models, whereas an early onset FAD mutation in PS1 results in constitute activation of CREB-phosphorylation which is associated with neurodegeneration [64–67] and BACE1 regulates the cAMP/PKA/CREB pathway independent of A*β* [68]. Thus, APP and components of its processing machinery impact neuronal signalling pathways independent of APP processing. Hence, FAD mutations can modulate signalling in potentially two ways: through elevated A*β*o formation via abnormal APP processing and/or independently of A*β* through altered interactions with signalling pathways. Perhaps through these combined effects on signalling such mutations represent particularly aggressive and penetrant forms of AD.

Considering that signalling and associated endocytosis is abnormal in FAD, it begs the question how this relates to sporadic AD. As amyloidogenic processing of APP is controlled by signalling and endocytosis, it is highly relevant to observe that AD risk factors, although very heterogeneous, have common mechanistic underpinnings by impacting intracellular signal transduction pathways (summarized in Table 1). For example, ApoE4 and traumatic brain injury, two entirely unrelated AD risk factors, both directly activate common signalling pathways (such as Erk1/2). In fact, for most nongenetic AD risk factors no direct impact on A*β* homeostasis can be hypothesized but involve altered signalling. Metabolic disorders like obesity or diabetes are associated with high levels of cytokines which activate AD relevant pathways in neurons. Likewise, glucocorticoids produced under conditions of chronic stress impact AD relevant signalling cascades in their own right. AD risk factors, such as stroke or head injury involve glutamate receptor-mediated excitotoxicity and impact Ca^2+^ signalling in a way which mechanistically resembles A*β*o-instigated activation of Erk1/2 by NMDA receptors. Ageing, the most prominent risk factor for AD, involves, apart from the abovementioned risk factors, altered redox signalling as a result of age-related decline of mitochondrial activity with concomitant increases in ROS production.

In summary, a common denominator in both FAD and sporadic AD comprises perturbation of intraneuronal signalling with associated changes in the endocytic pathway. As outlined above this may result in a vicious, self-enforcing cycle of deranged signalling and A*β* production driving the pathogenesis (Figure 1). In early onset FAD, this autocatalytic mechanism is directly and potently impacted by mutations in APP or its processing machinery. In late onset and sporadic AD initial, probably relatively minor, alterations of signalling by one or more AD risk factors may overtime set off this mechanism which once in motion drives AD pathogenesis.

This scenario resembles a domino system where tumbling of the stones (deranged signalling) is both cause and effect (autocatalytic effect), yet in order to let it happen a “risk factor” such as a sufficiently strong push, windfall, or vibration, is required to set off the cascade. To extent the metaphor further, FAD mutations could be seen as alterations of the core autocatalytic mechanism itself, for instance, as thinner domino stones, which make the system more unstable and thus more sensitive to risk factors. The opposite may be true for “protective” APP mutations (thicker stones, more resilient to risk factors) like the recently discovered Icelandic mutation which decreases APP processing [69].

From this perspective it can be envisaged that AD risk factors comprise a patient-specific constellation which determine the onset and progression of altered signalling and consequently AD pathogenesis. By extension any genetic, environmental, pharmacological, or lifestyle factor impacting this mechanism can, depending on the direction of the effect, be considered as a positive or negative AD risk factor.

### 2.4. A Signalling Function of Phosphorylated TAU Contributes to AD Pathogenesis

Besides A*β* polymerization and deposition into plaques, hyperphosphorylation and aggregation of TAU into intracellular tangles are other pathological features of AD. The identification of clinical mutations in TAU leading to FTLD strongly suggests that TAU in AD has an important role in pathogenesis [70–72]. Consistent with this notion, in many experimental paradigms a TAU-dependent neuronal degeneration was observed [23]. However, a key question remains as to the mechanism involved especially in relation to changes in signalling and A*β* homeostasis.

A study in transgenic APP mice, a model of early onset AD without TAU-tangle formation, revealed that deletion of the endogenous TAU mouse gene rescues cognitive decline without impacting plaque formation [73]. These findings position TAU as a downstream mediator required for APP-instigated neuronal toxicity, a feature not involving a loss-of-function (i.e., decreased microtubule stabilization), but a gain-of-toxic function which, however, does not involve TAU tangles [74]. Instead it was shown that TAU regulates postsynaptic NMDAR signalling directly by a mechanism involving recruitment of Src kinase Fyn to the PSD95-NMDA receptor complex [75, 76]. Combined with the observation that, like deletion of TAU, lowering of NMDAR-Erk1/2 signalling rescues APP-driven toxicity [75, 77] it appears that in AD such TAU function potentiates NMDA receptor signalling [76, 78]. Likewise, A*β*o activation of the mGluR5 receptor through PrP^C^ may also involve Fyn-TAU interaction [26, 79]. In other words TAU has, besides its well-known function in binding and stabilizing microtubules, a role in intracellular signalling. This raises the distinct possibility that when TAU's signalling activity goes awry it may contribute to AD pathogenesis.

Albeit TAU's signalling function is a somewhat neglected feature, a far more general role of TAU in signalling (apart from impacting NMDA receptors) can be considered.  Table 3 shows numerous TAU interactors which are transducers of receptor-mediated signalling implying that TAU can modify their activity through these interactions. These interactors function in a variety of pathways both pre- and postsynaptically. Indeed, apart from impacting postsynaptic NMDA receptor activity, TAU activates presynaptic growth factor signalling through interaction with Src family kinases [80–82] or phospholipid signalling by activation of PLC*γ* [83] and would provide a mechanistic explanation as to the role of TAU in neurite outgrowth [84, 85] and cell cycle reentry [86, 87] in cell line models.

From Table 3 it can also be appreciated that many interactors through their SH3 domains bind to the proline-rich domain (PRD) of TAU. Notably, TAU PRD is hyperphosphorylated in AD suggesting that these interactions are controlled by TAU-phosphorylation (and indirectly the relevant signalling cascades). Quantifying the TAU-SH3 interaction by surface plasmon resonance and sedimentation assays indicated this may indeed be the case [88, 89]. Phosphorylation-mimicking mutations of TAU were shown to increase or decrease (depending on the TAU-isoform) the affinity to Fyn or Src SH3 domains, consistent with the requirement of TAU-phosphorylation for regulation of NGF-RAS-Erk1/2 signalling [80]. Moreover, clinical FTLD-causing TAU-mutations were found to strongly increase the affinity to SH3, that is, phenocopying the effects of hyperphosphorylation [88]. Thus, these mutations could directly impact signalling, similar as in AD, which may contribute to neuronal degeneration. In fact, it may provide an explanation as to the mechanism of FTLD mutations in TAU which do not impact its aggregation propensity [90] such as R406W [91–94], which possesses an increased affinity to Fyn-SH3 of about 45 times [88]. Collectively, it seems possible that deregulation of signalling by hyperphosphorylated TAU constitutes a toxic gain-of-function of TAU driving pathogenesis in AD (Figure 1).

### 2.5. Deregulated Signalling by A*β*o or Other AD Risk Factors Triggers TAU-Hyperphosphorylation

An important question however remains as to how TAU becomes hyperphosphorylated in the first place. The facts that TAU is a substrate of many of the kinases operating in the pathways modulated by A*β*o (Table 2) or by AD risk factors or genes (Table 1) and that TAU is phosphorylated by neurons challenged with A*β*o or other stresses/conditions [95–98], provide a mechanistic explanation as to TAU hyperphosphorylation in AD [5, 23, 99, 100]. Once phosphorylated, TAU may impact intracellular signalling further implying a positive feedback mechanism such as that proposed for TAU-potentiated NGF-Erk1/2 activation [80] and/or by recruitment of Fyn to NMDA receptors [75].

Another salient feature entails the somatodendritic redistribution of TAU in diseased neurons, a prerequisite for impacting postsynaptic signalling. This feature of TAU is controlled by phosphorylation of microtubule binding repeat domains which strongly reduces its affinity to microtubules [101]. Hyperphosphorylation of TAU (and presumably detachment from microtubules) is a prerequisite—by an as yet unclear mechanism—to cross an axonal diffusion barrier allowing TAU to invade the somatodendritic space [102]. Phosphorylation of TAU at the repeat domains, in particular Ser262, is required to elicit A*β*-instigated neurotoxicity [95, 103, 104], indicating that detachment from microtubules entails an important feature of AD pathogenesis. Moreover, Ser262 is one of the earliest sites phosphorylated in the course of pathogenesis [22] and its phosphorylation acts as a priming site for further, more extensive phosphorylation at sites that may control its signalling function [105]. These results indicate a sequential mechanism of TAU-phosphorylation by A*β*o and other AD risk factors affecting its subcellular distribution and signalling.

Collectively TAU-phosphorylation comprises a gain-of-toxic function driving AD pathogenesis. Activation of signalling pathways by A*β*o and/or by other triggers (see Table 1) leads to hyperphosphorylation of TAU which subsequently decreases its microtubule binding and alters its somatodendritic redistribution and signalling function. These effects may be amplified by a feedback mechanism as formation of A*β*o and phosphorylated TAU not only control but are also controlled by signalling (Figure 1). The resulting deregulation of intraneuronal signalling contribute to neurodegeneration (see below), whereas the elevated levels of A*β*o and phosphorylated TAU, which have a high propensity to aggregate, lead to A*β* plaques and TAU tangles.

## 3. Considerations on the Mechanism of Altered Signalling in Neurodegeneration

### 3.1. Intraneuronal Signalling Defines Fate and Function of a Neuron for Better and for Worse

As discussed above deregulation of signalling may drive the neuronal degeneration in AD. The question, however, remains as to how mechanistically “deregulation” of pathways lead to neuronal degeneration. Neuronal function and survival depend on a balance between neurotrophic and neurotoxic cues setting off signalling cascades which define the outcome ranging from proliferation, differentiation, synaptic plasticity to apoptosis. A classical example entails growth factor signalling which sustains neuronal survival and can trigger differentiation or even antagonize the effects of toxic insults, whereas neurons without sufficient trophic support are prone to undergo apoptosis, as it occurs in a developing nervous system [106, 107]. Thus, properly regulated and balanced signalling, in function of its developmental state, defines fate and function of a neuron [108]. Accordingly, pathological conditions, such as in AD, which off-balance signalling are expected to decrease neuronal integrity.

The underlying mechanisms of how signalling leads to altered neuronal function are poorly understood but extensive work on Erk1/2 signalling revealed insights which may be applicable to other neuronal signalling pathways as well [109]. Erk1/2 signalling is particularly relevant for AD since its aberrant activation is an important driver of neurodegeneration [109, 110]. Erk1/2 kinases are responsive to a wide variety of functional (learning and memory), trophic, and pathogenic stimuli leading to different, even opposing, outcomes including survival, proliferation, differentiation, and neuronal cell death [110–112]. Thus, the signal as such is not predictive of the outcome and additional layers of control exist to determine specificity of Erk1/2 activation. Compartmentalization is a prominent mechanism to ensure specificity as it directs and concentrates the kinase (or sometimes the whole signalling pathway) to appropriate substrates within the cell [113]. This remarkable feature involves several scaffold, anchor, and retention factors which bind to Erk1/2 and often also other signalling molecules determining its subcellular action and allowing crosstalk with other pathways [113].

Localisation of Erk1/2 to specify its output is in part controlled by the kinetics of the signal (reviewed in [114, 115]). During transient activation, Erk1/2 remains predominantly cytoplasmic promoting proliferation, whereas its sustained activation is needed for nuclear concentration and results in differentiation. Chronic stress causes prolonged Erk1/2 activation in the nucleus which contributes to cell death [109, 116]. Thus, the widely different outcomes of Erk1/2 signalling depends, at least in part, on its kinetics as it dictates its subcellular localisation and as such specifies accessibility of substrates (reviewed in [113, 115]). In several model systems, sustained Erk1/2 activation involves a nuclear accumulation which is associated with detrimental outcomes [116–120]. For instance, neurons challenged with stress trigger a persistent nuclear retention of activated Erk1/2 and elicit proapoptotic effects and cell death [109, 116]. Accordingly, it seems likely that the chronic activation of Erk1/2 in AD, presumably by A*β*o and possibly other risk factors (see Tables 1 and 2), leads to an aberrant, prolonged nuclear accumulation contributing to neuronal demise.

### 3.2. Diseased Neurons in AD Display Signalling Configured for Immature Neurons

The insights obtained from the studies on Erk1/2 revealed that spatiotemporal control of Erk1/2 signalling determines its impact on neuronal function and survival [109] and as such provide a conceptual framework of the underlying mechanisms as to how derailed Erk1/2 signalling contributes to neuronal degeneration in AD. We anticipate that this concept is likely applicable to other signalling pathways as well.

In fact hyperphosphorylation of TAU in AD can be considered a reflection of such global deregulation of signalling in adult neurons [6] and illustrates how this may lead to inappropriate outcomes in function of the developmental state. As outlined above, hyperphosphorylation of TAU may lead to increased microtubule dynamics and the potentiating of pathways (such as Erk1/2) resulting in aberrant cell cycle entry and apoptosis. Such functional outcomes are expected to be detrimental in mature, postmitotic neurons of the adult brain. However, in a developing brain hyperphosphorylated TAU is fully appropriate as, in this context, neurons require dynamic microtubules to mediate sufficient synaptic plasticity, proliferation, and differentiation but also susceptibility to undergo apoptosis when trophic support by target cells is insufficient [108]. In other words, neuronal signalling in AD involving TAU-hyperphosphorylation appears to be geared to a situation resembling an immature brain.

Perhaps, a similar situation may apply for APP and its processing as well, given the neurotrophic properties of APP and its cleavage products [121]. Addition of APP to PC12 cells stimulates neurite outgrowth [122], whereas in transgenic mice expression of human APP results in increased neurogenesis [123, 124]. Moreover, the AICD promotes signalling associated with neurite outgrowth [18, 50], and secreted sAPP*α* impacts proliferation of embryonic stem cells [125]. Remarkably, at low concentration, A*β* has neurotrophic activity but only in undifferentiated neurons but is toxic to mature neurons [126–129]. Thus, APP and its processing products may have a role in proliferation and differentiation, functions that are particularly relevant in a developing brain, but, when unchecked, toxic to mature neurons.

Collectively it can be envisaged that APP, its processing products, and TAU are part of an intraneuronal signalling network required for neurogenesis, neuronal function, and survival which needs to be appropriately tuned to the developmental status. Accordingly, pathological conditions or risk factors which off-balance such signalling network to a state resembling immature neurons will be detrimental for mature neurons.

### 3.3. Considerations on Drug Discovery for Alzheimer's Disease

As discussed above aberrant activation of signalling cascades underlies mechanistically neurodegeneration in AD. As such it may provide a conceptual framework for successful drug discovery as it assumes that interventions aimed at normalizing signalling are expected to be neuroprotective, to reduce A*β* levels and TAU-phosphorylation and consequently plaque and tangle formation. In this way a fundamental mechanism driving pathogenesis in AD will be targeted and thus anticipates the minimum to preserve the function of still healthy neurons in the diseased brain and possibly may even restore dysfunctional synaptic activity of affected, but still living, neurons in symptomatic patients. However, given the multitude of pathways involved and considering their important neuronal functions, pharmacological modulation of one, specific target safely to achieve that goal will be a major challenge. Another confounding factor comprises the heterogeneity of sporadic patients, presumably reflected by the heterogeneity of risk factors each with their specific effects on the nature and effect size of the signalling pathways.

A*β*-directed therapeutic approaches to reduce A*β* levels have been and are still heavily explored and are expected to normalize signalling, at least to some extent, and thus have therapeutic potential. However, a possible downside may be that in symptomatic patients TAU-hyperphosphorylation has kicked in already to a level able to derange signalling and neuronal function in a feed forward fashion independent of A*β*o (from that point on perhaps mechanistically similar to how clinical TAU mutations in FTLD lead to neurodegeneration). Thus, such approach would be most successful in a preventive setup very early in the development of AD. Another consideration is that the therapeutic intervention itself should not inadvertently impact neuronal signalling for the worse. For instance, inhibiting *γ*-secretase will, on one hand, lead to lowered A*β*o levels and most likely to cognitive improvement in transgenic APP mouse models of familial AD but on the other hand may also impact signalling pathways (such as increased Erk1/2 activity [65]), independent of APP processing, which may impair a therapeutic response in sporadic AD patients. Likewise inhibition of CDK5, a prominent TAU-kinase and considered an attractive drug target for AD [130], may lead to sustained Erk1/2 activity and consequently neuronal apoptosis [131].

Nevertheless, promising drug targets to be considered for therapeutic intervention comprise components of signalling pathways impacted in AD [6, 130] although there is a risk—given the overall deregulation of signalling—that downregulation of only one kinase (or pathway) might be too limited to result in a satisfying therapeutic response. From this perspective, an interesting point of intervention may comprise the convergence where receptors relay their environmental cues to second messengers such as Ca^2+^ and/or small GTPases modules (Figure 1). Downregulating, but not fully inhibiting, the activity of such relay systems may lead to a more global normalization of signalling in AD and thus may constitute a promising therapeutic avenue.

## Figures and Tables

**Table 1:** Sporadic and familial AD risk genes and nongenetic positive risk factors and possible pathogenic mechanisms [8, 132].

Risk factor	Possible mechanism(s)∗		References∗∗
	A*β* homeostasis	Cellular signaling	
**Genetic**			
*APP *	APP processing	Erk1/2	[61]
*PS1 *	Change in A*β*40/A*β*42 ratio	Wnt-signalling, Erk1/2, Akt, and Ca^2+^ signaling	[61, 64–66, 133]
*PS2 *	APP processing	Erk1/2	[65, 134, 135]
*BACE *	APP processing	cAMP-PKA-CREB signaling	[68]
*ApoE4 *	A*β* clearance	Erk1/2, JNK	[136–140]
*SORLA *	APP processing	Neurotrophin signaling	[137, 141, 142]
*EPHA1 *	?	Ephrin signalling (Erk1/2)	[143, 144]
*MS4A6A/MS4A4A *	?	Signalling	[132]
*CD2AP *	?	PI3K-Akt-GSK3 (podocytes)	[145]
*CLU *	A*β* sequestering	Leptin/clusterin signalling; p53-Dkk1-JNK pathway	[146–148]
*β2-AR *	?	PKA, Erk1/2, and JNK	[149, 150]
*CD33 *	A*β* clearance		[151]
*PICALM *	APP processing	Regulation of receptor-mediated endocytosis?	[152]
*BIN1 *	APP processing	Ca^2+^ dyshomeostasis	[153]
*ABCA7 *	A*β* clearance	?	[154]

**Nongenetic**			
Smoking	?	Erk1/2 activation by oxidative stress	[155, 156]
Obesity	?	Cytokine-induced activation of MAPKs (p38, JNK); leptin signalling	[157–160]
Traumatic brain injury (TBI)	APP processing	Activation of MAPKs (Erk1/2, p38, and JNK), Akt, GSK3*β*	[8, 161]
Type II diabetes	?	Insulin signalling, cytokine-induced activation of MAPK's (p38, JNK)	[158–160, 162]
Stress (hormones)	?	Glucocorticoid-induced activation of Erk1/2, JNK; oxidative stress-induced JNK-dependent APP processing	[163–166]
Anaesthetics		Activation of MAPKs (Erk1/2, JNK)	[167–170]
Ageing	APP processing	Impaired Ca^2+^ dyshomeostasis and signalling, elevated cytokine signalling (“inflammaging”), impaired mitochondrial function with altered redox signalling (MAPKs, PI3K/Akt)	[171–174]

∗Not exhaustive. ∗∗Including reviews with original research papers cited.

**Table 2:** Neuronal receptors impacted by A*β*o [19, 175] and possible effects on downstream signalling pathways.

Receptor	Signal transduction pathway	References∗
NMDAR (NR2B subtype)	Erk1/2, CamKIV	[95, 176–182]
mGluR5 (with PrP^C^)	PKC, MAPKs (Erk1/2, p38, and JNK)	[79]
nAchR (*α*7 subtype)	Erk1/2, Akt, and JAK-STAT	[183, 184]
Wnt receptor	Wnt signalling (GSK3)	[185, 186]
IR/IGF	PI3K-Akt	[176, 187]
Amylin receptor	Erk1/2, PKA	[177]
RAGE	p38	[188]
Neurotrophin receptors	Erk1/2, Akt	[45, 189]
*β*2AR	PKA, Erk1/2, and JNK	[149, 190, 191]

∗Including reviews with original research papers cited.

**Table 3:** Binding partners of TAU (modified from [4, 192]).

Binding partner	Region of TAU involved	Function/identity of binding partner	References
*β*-tubulin	Repeat domains	Cytoskeleton	[193]
F-actin		Cytoskeleton	[194]
ApoE3	Repeat domains	Lipid carrier	[195, 196]
Fgr	Proline-rich domain	Src kinase family	[89]
Fyn	Proline-rich domain	Src kinase family	[82, 89]
Lck	Proline-rich domain	Src kinase family	[82, 89]
cSrc	Proline-rich domain	Src kinase family	[82, 89]
Grb2	Proline-rich domain	Growth factor signalling	[89]
c-Abl		Src kinase family	[197]
p85*α*	Proline-rich domain	Regulator PI3K, phospholipid signalling	[89]
PLC*γ*		Phospholipid signalling	[89, 198]
GSK3*β*	N-terminal	Kinase	[199]
Calmodulin	Repeat domain	Ca^2+^ signalling	[200, 201]
14-3-3	Proline-rich domain and repeat domain	Signalling scaffold	[202–204]
Annexin A2		Ca^2+^ signalling, membrane trafficking	[205]
Pin1	Proline-rich domain	Peptidyl-prolyl cis/trans isomerase regulates phosphorylation of TAU	[206, 207]

## References

[1] Selkoe DJ (2002). Alzheimer's disease is a synaptic failure. *Science*.

[2] Selkoe DJ (2011). Alzheimer's disease. *Cold Spring Harbor Perspectives in Biology*.

[3] O'Brien RJ, Wong PC (2011). Amyloid precursor protein processing and alzheimer's disease. *Annual Review of Neuroscience*.

[4] Morris M, Maeda S, Vossel K, Mucke L (2011). The many faces of tau. *Neuron*.

[5] Noble W, Hanger DP, Miller CC, Lovestone S (2013). The importance of tau phosphorylation for neurodegenerative diseases. *Frontiers in Neurology*.

[6] Martin L, Latypova X, Wilson CM (2013). Tau protein kinases: involvement in Alzheimer's disease. *Ageing Research Reviews*.

[7] Liu C, Kanekiyo T, Xu H, Bu G (2013). Apolipoprotein e and Alzheimer disease: risk, mechanisms and therapy. *Nature Reviews Neurology*.

[8] Mayeux R, Stern Y (2012). Epidemiology of Alzheimer disease. *Cold Spring Harbor Perspectives in Medicine*.

[9] Brouillette J, Caillierez R, Zommer N (2012). Neurotoxicity and memory deficits induced by soluble low-molecular-weight amyloid-*β*1-42 oligomers are revealed in vivo by using a novel animal model. *Journal of Neuroscience*.

[10] Forloni G, Balducci C (2011). *β*-amyloid oligomers and prion protein: fatal attraction?. *Prion*.

[11] Itkin A, Dupres V, Dufrêne YF, Bechinger B, Ruysschaert J, Raussens V (2011). Calcium ions promote formation of amyloid *β*-peptide (1-40) oligomers causally implicated in neuronal toxicity of Alzheimer's disease. *PLoS ONE*.

[12] Lublin AL, Gandy S (2010). Amyloid-*β* oligomers: possible roles as key neurotoxins in Alzheimer's disease. *Mount Sinai Journal of Medicine*.

[13] Miñano-Molina AJ, España J, Martín E (2011). Soluble oligomers of amyloid-*β* peptide disrupt membrane trafficking of α-amino-3-hydroxy-5-methylisoxazole-4-propionic acid receptor contributing to early synapse dysfunction. *The Journal of Biological Chemistry*.

[14] Ono K, Yamada M (2011). Low-n oligomers as therapeutic targets of Alzheimer's disease. *Journal of Neurochemistry*.

[15] Umeda T, Tomiyama T, Sakama N (2011). Intraneuronal amyloid *β* oligomers cause cell death via endoplasmic reticulum stress, endosomal/lysosomal leakage, and mitochondrial dysfunction in vivo. *Journal of Neuroscience Research*.

[16] Jiang Y, Mullaney KA, Peterhoff CM (2010). Alzheimer's-related endosome dysfunction in Down syndrome is A*β*-independent but requires APP and is reversed by BACE-1 inhibition. *Proceedings of the National Academy of Sciences of the United States of America*.

[17] Ghosal K, Vogt DL, Liang M, Shen Y, Lamb BT, Pimplikar SW (2009). Alzheimer's disease-like pathological features in transgenic mice expressing the APP intracellular domain. *Proceedings of the National Academy of Sciences of the United States of America*.

[18] Schettini G, Govoni S, Racchi M, Rodriguez G (2010). Phosphorylation of APP-CTF-AICD domains and interaction with adaptor proteins: signal transduction and/or transcriptional role—relevance for Alzheimer pathology. *Journal of Neurochemistry*.

[19] Patel AN, Jhamandas JH (2012). Neuronal receptors as targets for the action of amyloid-beta protein (A*β*) in the brain. *Expert Reviews in Molecular Medicine*.

[20] Hong S, Ostaszewski BL, Yang T (2014). Soluble A*β* oligomers are rapidly sequestered from brain ISF in vivo and bind GM1 ganglioside on cellular membranes. *Neuron*.

[21] Ledeen RW, Wu G (2002). Ganglioside function in calcium homeostasis and signaling. *Neurochemical Research*.

[22] Augustinack JC, Schneider A, Mandelkow E, Hyman BT (2002). Specific tau phosphorylation sites correlate with severity of neuronal cytopathology in Alzheimer's disease. *Acta Neuropathologica*.

[23] Stoothoff WH, Johnson GVW (2005). Tau phosphorylation: physiological and pathological consequences. *Biochimica et Biophysica Acta: Molecular Basis of Disease*.

[24] Thathiah A, De Strooper B (2011). The role of G protein-coupled receptors in the pathology of Alzheimer's disease. *Nature Reviews Neuroscience*.

[25] Thathiah A, Spittaels K, Hoffmann M (2009). The orphan G protein-coupled receptor 3 modulates amyloid-beta peptide generation in neurons. *Science*.

[26] Mairet-Coello G, Polleux F (2014). Involvement of ‘stress-response’ kinase pathways in Alzheimer's disease progression. *Current Opinion in Neurobiology*.

[27] Yoon SO, Park DJ, Ryu JC (2012). JNK3 perpetuates metabolic stress induced by A*β* peptides. *Neuron*.

[28] Ruiz-León Y, Pascual A (2004). Regulation of *β*-amyloid precursor protein expression by brain-derived neurotrophic factor involves activation of both the Ras and phosphatidylinositide 3-kinase signalling pathways. *Journal of Neurochemistry*.

[29] Mitsuda N, Ohkubo N, Tamatani M (2001). Activated cAMP-response element-binding protein regulates neuronal expression of presenilin-1. *Journal of Biological Chemistry*.

[30] Mizuno S, Iijima R, Ogishima S (2012). AlzPathway: a comprehensive map of signaling pathways of Alzheimer's disease. *BMC Systems Biology*.

[31] Sorkin A, Von Zastrow M (2009). Endocytosis and signalling: intertwining molecular networks. *Nature Reviews Molecular Cell Biology*.

[32] Murphy JE, Padilla BE, Hasdemir B, Cottrell GS, Bunnett NW (2009). Endosomes: a legitimate platform for the signaling train. *Proceedings of the National Academy of Sciences of the United States of America*.

[33] Dobrowolski R, de Robertis EM (2012). Endocytic control of growth factor signalling: multivesicular bodies as signalling organelles. *Nature Reviews Molecular Cell Biology*.

[34] Zweifel LS, Kuruvilla R, Ginty DD (2005). Functions and mechanisms of retrograde neurotrophin signalling. *Nature Reviews Neuroscience*.

[35] Ascano M, Bodmer D, Kuruvilla R (2012). Endocytic trafficking of neurotrophins in neural development. *Trends in Cell Biology*.

[36] Platta HW, Stenmark H (2011). Endocytosis and signaling. *Current Opinion in Cell Biology*.

[37] Kikuchi A, Yamamoto H, Sato A (2009). Selective activation mechanisms of Wnt signaling pathways. *Trends in Cell Biology*.

[38] Di Paolo G, De Camilli P (2006). Phosphoinositides in cell regulation and membrane dynamics. *Nature*.

[39] Nixon RA (2005). Endosome function and dysfunction in Alzheimer's disease and other neurodegenerative diseases. *Neurobiology of Aging*.

[40] Cataldo AM, Peterhoff CM, Troncoso JC, Gomez-Isla T, Hyman BT, Nixon RA (2000). Endocytic pathway abnormalities precede amyloid *β* deposition in sporadic alzheimer's disease and down syndrome: differential effects of APOE genotype and presenilin mutations. *The American Journal of Pathology*.

[41] Ginsberg SD, Alldred MJ, Counts SE (2010). Microarray analysis of hippocampal CA1 neurons implicates early endosomal dysfunction during Alzheimer's disease progression. *Biological Psychiatry*.

[42] Nagele RG, D'Andrea MR, Anderson WJ, Wang H-Y (2002). Intracellular accumulation of *β*-amyloid1-42 in neurons is facilitated by the α7 nicotinic acetylcholine receptor in Alzheimer's disease. *Neuroscience*.

[43] Goto Y, Niidome T, Akaike A, Kihara T, Sugimoto H (2006). Amyloid *β*-peptide preconditioning reduces glutamate-induced neurotoxicity by promoting endocytosis of NMDA receptor. *Biochemical and Biophysical Research Communications*.

[44] Wang D, Yuen EY, Zhou Y, Yan Z, Xiang YK (2011). Amyloid *β* peptide-(1 - 42) induces internalization and degradation of *β*2 adrenergic receptors in prefrontal cortical neurons. *Journal of Biological Chemistry*.

[45] Bulbarelli A, Lonati E, Cazzaniga E (2009). TrkA pathway activation induced by amyloid-beta (Abeta). *Molecular and Cellular Neuroscience*.

[46] Stöhr O, Schilbach K, Moll L (2013). Insulin receptor signaling mediates APP processing and *β*-amyloid accumulation without altering survival in a transgenic mouse model of Alzheimer’s disease. *Age*.

[47] O'Neill C (2013). PI3-kinase/Akt/mTOR signaling: impaired on/off switches in aging, cognitive decline and Alzheimer's disease. *Experimental Gerontology*.

[48] Griffin RJ, Moloney A, Kelliher M (2005). Activation of Akt/PKB, increased phosphorylation of Akt substrates and loss and altered distribution of Akt and PTEN are features of Alzheimer's disease pathology. *Journal of Neurochemistry*.

[49] Cavalli V, Vilbois F, Corti M (2001). The stress-induced MAP kinase p38 regulates endocytic trafficking via the GDI:Rab5 complex. *Molecular Cell*.

[50] Zhou F, Gong K, Song B (2012). The APP intracellular domain (AICD) inhibits Wnt signalling and promotes neurite outgrowth. *Biochimica et Biophysica Acta*.

[51] Cirrito JR, Kang J, Lee J (2008). Endocytosis is required for synaptic activity-dependent release of amyloid-*β* in vivo. *Neuron*.

[52] Yu C, Nwabuisi-Heath E, Laxton K, Ladu MJ (2010). Endocytic pathways mediating oligomeric A*β*42 neurotoxicity. *Molecular Neurodegeneration*.

[53] Small SA, Gandy S (2006). Sorting through the cell biology of Alzheimer's disease: intracellular pathways to pathogenesis. *Neuron*.

[54] Wu J, Petralia RS, Kurushima H (2011). Arc/Arg3.1 Regulates an endosomal pathway essential for activity-dependent *β*-amyloid generation. *Cell*.

[55] Udayar V, Buggia-Prevot V, Guerreiro RL (2013). A Paired RNAi and RabGAP overexpression screen identifies Rab11 as a regulator of beta-amyloid production. *Cell Reports*.

[56] Das U, Scott DA, Ganguly A, Koo EH, Tang Y, Roy S (2013). Activity-induced convergence of app and BACE-1 in acidic microdomains via an endocytosis-dependent pathway. *Neuron*.

[57] Carey RM, Balcz BA, Lopez-Coviella I, Slack BE (2005). Inhibition of dynamin-dependent endocytosis increases shedding of the amyloid precursor protein ectodomain and reduces generation of amyloid beta protein. *BMC Cell Biology*.

[58] Buggia-Prevot V, Fernandez CG, Udayar V (2013). A function for EHD family proteins in unidirectional retrograde dendritic transport of BACE1 and Alzheimer's disease Abeta production. *Cell Reports*.

[59] Sannerud R, Declerck I, Peric A (2011). ADP ribosylation factor 6 (ARF6) controls amyloid precursor protein (APP) processing by mediating the endosomal sorting of BACE1. *Proceedings of the National Academy of Sciences of the United States of America*.

[60] Ulery PG, Beers J, Mikhailenko I (2000). Modulation of *β*-amyloid precursor protein processing by the low density lipoprotein receptor-related protein (LRP). Evidence that LRP contributes to the pathogenesis of Alzheimer's disease. *Journal of Biological Chemistry*.

[61] Nizzari M, Thellung S, Corsaro A (2012). Neurodegeneration in Alzheimer disease: role of amyloid precursor protein and presenilin 1 intracellular signaling. *Journal of Toxicology*.

[62] Vetrivel KS, Zhang Y, Xu H, Thinakaran G (2006). Pathological and physiological functions of presenilins. *Molecular Neurodegeneration*.

[63] Haass C, de Strooper B (1999). The presenilins in Alzheimer's disease—proteolysis holds the key. *Science*.

[64] Müller M, Cárdenas C, Mei L, Cheung K, Foskett JK (2011). Constitutive cAMP response element binding protein (CREB) activation by Alzheimer's disease presenilin-driven inositol trisphosphate receptor (InsP3R) Ca2+ signaling. *Proceedings of the National Academy of Sciences of the United States of America*.

[65] Dehvari N, Isacsson O, Winblad B, Cedazo-Minguez A, Cowburn RF (2008). Presenilin regulates extracellular regulated kinase (Erk) activity by a protein kinase C alpha dependent mechanism. *Neuroscience Letters*.

[66] Baki L, Shioi J, Wen P (2004). PS1 activates PI3K thus inhibiting GSK-3 activity and tau overphosphorylation: effects of FAD mutations. *The EMBO Journal*.

[67] Nizzari M, Venezia V, Repetto E (2007). Amyloid precursor protein and presenilin1 interact with the adaptor GRB2 and modulate ERK1,2 signaling. *The Journal of Biological Chemistry*.

[68] Chen Y, Huang X, Zhang Y (2012). Alzheimer's *β*-secretase (BACE1) regulates the cAMP/PKA/CREB pathway independently of *β*-amyloid. *The Journal of Neuroscience*.

[69] Jonsson T, Atwal JK, Steinberg S (2012). A mutation in APP protects against Alzheimer's disease and age-related cognitive decline. *Nature*.

[70] Hutton M, Lendon CL, Rizzu P (1998). Association of missense and 5′-splice-site mutations in tau with the inherited dementia FTDP-17. *Nature*.

[71] Goedert M, Crowther RA, Spillantini MG (1998). Tau mutations cause frontotemporal dementias. *Neuron*.

[72] Spillantini MG, Murrell JR, Goedert M, Farlow MR, Klug A, Ghetti B (1998). Mutation in the tau gene in familial multiple system tauopathy with presenile dementia. *Proceedings of the National Academy of Sciences of the United States of America*.

[73] Roberson ED, Scearce-Levie K, Palop JJ (2007). Reducing endogenous tau ameliorates amyloid *β*-induced deficits in an Alzheimer's disease mouse model. *Science*.

[74] Santacruz K, Lewis J, Spires T (2005). Tau suppression in a neurodegenerative mouse model improves memory function. *Science*.

[75] Ittner LM, Ke YD, Delerue F (2010). Dendritic function of tau mediates amyloid-*β* toxicity in Alzheimer’s disease mouse models. *Cell*.

[76] Mondragón-Rodríguez S, Trillaud-Doppia E, Dudilot A (2012). Interaction of endogenous tau protein with synaptic proteins is regulated by N-methyl-D-aspartate receptor-dependent tau phosphorylation. *Journal of Biological Chemistry*.

[77] Um JW, Nygaard HB, Heiss JK (2012). Alzheimer amyloid-*β* oligomer bound to postsynaptic prion protein activates Fyn to impair neurons. *Nature Neuroscience*.

[78] Amadoro G, Ciotti MT, Costanzi M, Cestari V, Calissano P, Canu N (2006). NMDA receptor mediates tau-induced neurotoxicity by calpain and ERK/MAPK activation. *Proceedings of the National Academy of Sciences of the United States of America*.

[79] Um JW, Kaufman AC, Kostylev M (2013). Metabotropic glutamate receptor 5 is a coreceptor for Alzheimer a*β* oligomer bound to cellular prion protein. *Neuron*.

[80] Leugers CJ, Lee G (2010). Tau potentiates nerve growth factor-induced mitogen-activated protein kinase signaling and neurite initiation without a requirement for microtubule binding. *Journal of Biological Chemistry*.

[81] Sharma VM, Litersky JM, Bhaskar K, Lee G (2007). Tau impacts on growth-factor-stimulated actin remodeling. *Journal of Cell Science*.

[82] Lee G, Todd Newman S, Gard DL, Band H, Panchamoorthy G (1998). Tau interacts with src-family non-receptor tyrosine kinases. *Journal of Cell Science*.

[83] Hwang SC, Jhon D, Bae YS, Kim JH, Rhee SG (1996). Activation of phospholipase C-*γ* by the concerted action of tau proteins and arachidonic acid. *The Journal of Biological Chemistry*.

[84] Yoshizaki C, Tsukane M, Yamauchi T (2004). Overexpression of tau leads to the stimulation of neurite outgrowth, the activation of caspase 3 activity, and accumulation and phosphorylation of tau in neuroblastoma cells on cAMP treatment. *Neuroscience Research*.

[85] Biernat J, Wu Y, Timm T (2002). Protein kinase MARK/PAR-1 is required for neurite outgrowth and establishment of neuronal polarity. *Molecular Biology of the Cell*.

[86] Seward ME, Swanson E, Norambuena A (2013). Amyloid-*β* signals through tau to drive ectopic neuronal cell cycle re-entry in alzheimer's disease. *Journal of Cell Science*.

[87] Hoerndli FJ, Pelech S, Papassotiropoulos A, Götz J (2007). A*β* treatment and P301L tau expression in an Alzheimer's disease tissue culture model act synergistically to promote aberrant cell cycle re-entry. *European Journal of Neuroscience*.

[88] Bhaskar K, Yen S, Lee G (2005). Disease-related modifications in tau affect the interaction between Fyn and tau. *Journal of Biological Chemistry*.

[89] Reynolds CH, Garwood CJ, Wray S (2008). Phosphorylation regulates tau interactions with Src homology 3 domains of phosphatidylinositol 3-kinase, phospholipase C*γ*1, Grb2, and Src family kinases. *The Journal of Biological Chemistry*.

[90] Brandt R, Hundelt M, Shahani N (2005). Tau alteration and neuronal degeneration in tauopathies: mechanisms and models. *Biochimica et Biophysica Acta*.

[91] Goedert M, Jakes R, Crowther RA (1999). Effects of frontotemporal dementia FTDP-17 mutations on heparin-induced assembly of tau filaments. *FEBS Letters*.

[92] Deture M, Ko L, Yen S (2000). Missense tau mutations identified in FTDP-17 have a small effect on tau-microtubule interactions. *Brain Research*.

[93] Nacharaju P, Lewis J, Easson C (1999). Accelerated filament formation from tau protein with specific FTDP-17 missense mutations. *FEBS Letters*.

[94] Goedert M, Spillantini MG (2000). Tau mutations in frontotemporal dementia FTDP-17 and their relevance for Alzheimer's disease. *Biochimica et Biophysica Acta—Molecular Basis of Disease*.

[95] Mairet-Coello G, Courchet J, Pieraut S, Courchet V, Maximov A, Polleux F (2013). The CAMKK2-AMPK kinase pathway mediates the synaptotoxic effects of Abeta oligomers through Tau phosphorylation. *Neuron*.

[96] Jin M, Shepardson N, Yang T, Chen G, Walsh D, Selkoe DJ (2011). Soluble amyloid *β*-protein dimers isolated from Alzheimer cortex directly induce Tau hyperphosphorylation and neuritic degeneration. *Proceedings of the National Academy of Sciences of the United States of America*.

[97] Zempel H, Thies E, Mandelkow E (2010). A*β* oligomers cause localized Ca^2+^ elevation, missorting of endogenous Tau into dendrites, Tau phosphorylation, and destruction of microtubules and spines. *The Journal of Neuroscience*.

[98] Rapoport M, Dawson HN, Binder LI, Vitek MP, Ferreira A (2002). Tau is essential to *β*-amyloid-induced neurotoxicity. *Proceedings of the National Academy of Sciences of the United States of America*.

[99] Hanger DP, Anderton BH, Noble W (2009). Tau phosphorylation: the therapeutic challenge for neurodegenerative disease. *Trends in Molecular Medicine*.

[100] Lee G, Leugers CJ (2012). Tau and tauopathies. *Progress in Molecular Biology and Translational Science*.

[101] Biernat J, Gustke N, Drewes G, Mandelkow E (1993). Phosphorylation of Ser262 strongly reduces binding of tau to microtubules: Distinction between PHF-like immunoreactivity and microtubule binding. *Neuron*.

[102] Li X, Kumar Y, Zempel H, Mandelkow E, Biernat J, Mandelkow E (2011). Novel diffusion barrier for axonal retention of Tau in neurons and its failure in neurodegeneration. *EMBO Journal*.

[103] Iijima K, Gatt A, Iijima-Ando K (2010). Tau Ser262 phosphorylation is critical for A*β*42-induced tau toxicity in a transgenic Drosophila model of Alzheimer's disease. *Human Molecular Genetics*.

[104] Yu W, Polepalli J, Wagh D, Rajadas J, Malenka R, Lu B (2012). A critical role for the PAR-1/MARK-tau axis in mediating the toxic effects of A*β* on synapses and dendritic spines. *Human Molecular Genetics*.

[105] Bertrand J, Plouffe V, Sénéchal P, Leclerc N (2010). The pattern of human tau phosphorylation is the result of priming and feedback events in primary hippocampal neurons. *Neuroscience*.

[106] Twiss JL, Chang JH, Schanen NC (2006). Pathophysiological mechanisms for actions of the neurotrophins. *Brain Pathology*.

[107] Bibel M, Barde Y (2000). Neurotrophins: Key regulators of cell fate and cell shape in the vertebrate nervous system. *Genes and Development*.

[108] Kole AJ, Annis RP, Deshmukh M (2013). Mature neurons: equipped for survival. *Cell Death & Disease*.

[109] Colucci-D'Amato L, Perrone-Capano C, di Porzio U (2003). Chronic activation of ERK and neurodegenerative diseases. *BioEssays*.

[110] Zhu X, Lee H, Raina AK, Perry G, Smith MA (2002). The role of mitogen-activated protein kinase pathways in Alzheimer’s disease. *NeuroSignals*.

[111] Thomas GM, Huganir RL (2004). MAPK cascade signalling and synaptic plasticity. *Nature Reviews Neuroscience*.

[112] Grewal SS, York RD, Stork PJ (1999). Extracellular-signal-regulated kinase signalling in neurons. *Current Opinion in Neurobiology*.

[113] Wortzel I, Seger R (2011). The ERK cascade: distinct functions within various subcellular organelles. *Genes and Cancer*.

[114] Marshall CJ (1995). Specificity of receptor tyrosine kinase signaling: Transient versus sustained extracellular signal-regulated kinase activation. *Cell*.

[115] Caunt CJ, Keyse SM (2013). Dual-specificity MAP kinase phosphatases (MKPs): shaping the outcome of MAP kinase signalling. *FEBS Journal*.

[116] Stanciu M, Defranco DB (2002). Prolonged nuclear retention of activated extracellular signal-regulated protein kinase promotes cell death generated by oxidative toxicity or proteasome inhibition in a neuronal cell line. *Journal of Biological Chemistry*.

[117] Subramaniam S, Strelau J, Unsicker K (2003). Growth differentiation factor-15 prevents low potassium-induced cell death of cerebellar granule neurons by differential regulation of Akt and ERK pathways. *The Journal of Biological Chemistry*.

[118] Kulich SM, Chu CT (2001). Sustained extracellular signal-regulated kinase activation by 6-hydroxydopamine: implications for Parkinson’s disease. *Journal of Neurochemistry*.

[119] Stanciu M, Wang Y, Kentor R (2000). Persistent activation of ERK contributes to glutamate-induced oxidative toxicity in a neuronal cell line and primary cortical neuron cultures. *The Journal of Biological Chemistry*.

[120] Jiang Q, Gu Z, Zhang G (2001). Nuclear translocation of extracellular signal-regulated kinases in neuronal excitotoxicity. *NeuroReport*.

[121] Gralle M, Ferreira ST (2007). Structure and functions of the human amyloid precursor protein: the whole is more than the sum of its parts. *Progress in Neurobiology*.

[122] Milward EA, Papadopoulos R, Fuller SJ (1992). The amyloid protein precursor of Alzheimer's disease is a mediator of the effects of nerve growth factor on neurite outgrowth. *Neuron*.

[123] Jin K, Galvan V, Xie L (2004). Enhanced neurogenesis in Alzheimer's disease transgenic (PDGF-APP Sw,Ind) mice. *Proceedings of the National Academy of Sciences of the United States of America*.

[124] Yu Y, He J, Zhang Y (2009). Increased hippocampal neurogenesis in the progressive stage of Alzheimer's disease phenotype in an APP/PS1 double transgenic mouse model. *Hippocampus*.

[125] Porayette P, Gallego MJ, Kaltcheva MM, Bowen RL, Meethal SV, Atwood CS (2009). Differential processing of amyloid-*β* precursor protein directs human embryonic stem cell proliferation and differentiation into neuronal precursor cells. *The Journal of Biological Chemistry*.

[126] López-Toledano MA, Shelanski ML (2004). Neurogenic effect of *β*-amyloid peptide in the development of neural stem cells. *The Journal of Neuroscience*.

[127] Heo C, Chang K, Choi HS (2007). Effects of the monomeric, oligomeric, and fibrillar A*β*42 peptides on the proliferation and differentiation of adult neural stem cells from subventricular zone. *Journal of Neurochemistry*.

[128] Calafiore M, Battaglia G, Zappalà A (2006). Progenitor cells from the adult mouse brain acquire a neuronal phenotype in response to *β*-amyloid. *Neurobiology of Aging*.

[129] Yankner BA, Duffy LK, Kirschner DA (1990). Neurotrophic and neurotoxic effects of amyloid *β* protein: reversal by tachykinin neuropeptides. *Science*.

[130] Mazanetz MP, Fischer PM (2007). Untangling tau hyperphosphorylation in drug design for neurodegenerative diseases. *Nature Reviews Drug Discovery*.

[131] Zheng Y, Li B, Kanungo J (2007). Cdk5 modulation of mitogen-activated protein kinase signaling regulates neuronal survival. *Molecular Biology of the Cell*.

[132] Tanzi RE (2012). The genetics of Alzheimer disease. *Cold Spring Harbor Perspectives in Medicine*.

[133] Wolfe MS (2007). When loss is gain: reduced presenilin proteolytic function leads to increased A*β*42/A*β*40. Talking Point on the role of presenilin mutations in Alzheimer disease. *EMBO Reports*.

[134] Park MH, Choi DY, Jin HW (2012). Mutant presenilin 2 increases *β*-secretase activity through reactive oxygen species-dependent activation of extracellular signal-regulated kinase. *Journal of Neuropathology & Experimental Neurology*.

[135] Kang DE, Yoon IS, Repetto E (2005). Presenilins mediate phosphatidylinositol 3-kinase/AKT and ERK activation via select signaling receptors: selectivity of PS2 in platelet-derived growth factor signaling. *The Journal of Biological Chemistry*.

[136] Korwek KM, Trotter JH, Ladu MJ, Sullivan PM, Weeber EJ (2009). Apoe isoform-dependent changes in hippocampal synaptic function. *Molecular Neurodegeneration*.

[137] Bu G (2009). Apolipoprotein e and its receptors in Alzheimer's disease: حathways, pathogenesis and therapy. *Nature Reviews Neuroscience*.

[138] Lleó A, Waldron E, Von Arnim CAF (2005). Low density lipoprotein receptor-related protein (LRP) interacts with presenilin 1 and is a competitive substrate of the amyloid precursor protein (APP) for *γ*-secretase. *The Journal of Biological Chemistry*.

[139] DeMattos RB, Cirrito JR, Parsadanian M (2004). ApoE and clusterin cooperatively suppress Abeta levels and deposition: evidence that ApoE regulates extracellular Abeta metabolism in vivo. *Neuron*.

[140] Harris FM, Brecht WJ, Xu Q, Mahley RW, Huang Y (2004). Increased tau phosphorylation in apolipoprotein E4 transgenic mice is associated with activation of extracellular signal-regulated kinase: modulation by zinc. *The Journal of Biological Chemistry*.

[141] Willnow TE, Carlo A, Rohe M, Schmidt V (2010). SORLA/SORL1, a neuronal sorting receptor implicated in Alzheimer's disease. *Reviews in the Neurosciences*.

[142] Glerup S, Lume M, Olsen D (2013). SorLA controls neurotrophic activity by sorting of GDNF and its receptors GFRα1 and RET. *Cell Reports*.

[143] Nie D, di Nardo A, Han JM (2010). Tsc2-Rheb signaling regulates EphA-mediated axon guidance. *Nature Neuroscience*.

[144] Elowe S, Holland SJ, Kulkarni S, Pawson T (2001). Downregulation of the Ras-mitogen-activated protein kinase pathway by the EphB2 receptor tyrosine kinase is required for ephrin-induced neurite retraction. *Molecular and Cellular Biology*.

[145] Huber TB, Hartleben B, Kim J (2003). Nephrin and CD2AP associate with phosphoinositide 3-OH kinase and stimulate AKT-dependent signaling. *Molecular and Cellular Biology*.

[146] Killick R, Ribe EM, Al-Shawi R (2012). Clusterin regulates *β*-amyloid toxicity via Dickkopf-1-driven induction of the wnt-PCP-JNK pathway. *Molecular Psychiatry*.

[147] Gil SY, Youn B, Byun K (2013). Clusterin and LRP2 are critical components of the hypothalamic feeding regulatory pathway. *Nature Communications*.

[148] Narayan P, Orte A, Clarke RW (2012). The extracellular chaperone clusterin sequesters oligomeric forms of the amyloid-*β* 1-40 peptide. *Nature Structural and Molecular Biology*.

[149] Wang D, Fu Q, Zhou Y (2013). *Β*2 adrenergic receptor, protein kinase a (PKA) and c-Jun N-terminal kinase (JNK) signaling pathways mediate tau pathology in alzheimer disease models. *The Journal of Biological Chemistry*.

[150] Yu JT, Tan L, Ou J (2008). Polymorphisms at the *β*2-adrenergic receptor gene influence Alzheimer’s disease susceptibility. *Brain Research*.

[151] Griciuc A, Serrano-Pozo A, Parrado AR (2013). Alzheimer's disease risk gene cd33 inhibits microglial uptake of amyloid beta. *Neuron*.

[152] Xiao Q, Gil S, Yan P (2012). Role of Phosphatidylinositol Clathrin Assembly Lymphoid-Myeloid Leukemia (PICALM) in intracellular Amyloid Precursor Protein (APP) processing and amyloid plaque pathogenesis. *Journal of Biological Chemistry*.

[153] Tan MS, Yu JT, Tan L (2013). Bridging integrator 1 (BIN1): form, function, and Alzheimer's disease. *Trends in Molecular Medicine*.

[154] Kim WS, Li H, Ruberu K (2013). Deletion of Abca7 increases cerebral amyloid-*β* accumulation in the J20 mouse model of Alzheimer's disease. *Journal of Neuroscience*.

[155] Chu CT, Levinthal DJ, Kulich SM, Chalovich EM, DeFranco DB (2004). Oxidative neuronal injury: the dark side of ERK1/2. *European Journal of Biochemistry*.

[156] Ho Y-S, Yang X, Yeung S-C (2012). Cigarette smoking accelerated brain aging and induced pre-alzheimer-like neuropathology in rats. *PLoS ONE*.

[157] Greco SJ, Sarkar S, Johnston JM, Tezapsidis N (2009). Leptin regulates tau phosphorylation and amyloid through AMPK in neuronal cells. *Biochemical and Biophysical Research Communications*.

[158] Mehan S, Meena H, Sharma D, Sankhla R (2011). JNK: a stress-activated protein kinase therapeutic strategies and involvement in Alzheimer’s and various neurodegenerative abnormalities. *Journal of Molecular Neuroscience*.

[159] Correa SA, Eales KL (2012). The role of p38 MAPK and its substrates in neuronal plasticity and neurodegenerative disease. *Journal of Signal Transduction*.

[160] Quintanilla RA, Orellana DI, González-Billault C, Maccioni RB (2004). Interleukin-6 induces Alzheimer-type phosphorylation of tau protein by deregulating the cdk5/p35 pathway. *Experimental Cell Research*.

[161] Neary JT (2005). Protein kinase signaling cascades in CNS trauma. *IUBMB Life*.

[162] Schiöth HB, Craft S, Brooks SJ, Frey WH, Benedict C (2012). Brain insulin signaling and Alzheimer's disease: current evidence and future directions. *Molecular Neurobiology*.

[163] Sotiropoulos I, Catania C, Pinto LG (2011). Stress acts cumulatively to precipitate Alzheimer's disease-like tau pathology and cognitive deficits. *Journal of Neuroscience*.

[164] Rissman RA, Lee K, Vale W, Sawchenko PE (2007). Corticotropin-releasing factor receptors differentially regulate stress-induced tau phosphorylation. *Journal of Neuroscience*.

[165] Xia M, Hyman BT (2002). GROα/KC, a chemokine receptor CXCR2 ligand, can be a potent trigger for neuronal ERK1/2 and PI-3 kinase pathways and for tau hyperphosphorylation: a role in Alzheimer's disease?. *Journal of Neuroimmunology*.

[166] Quiroz-Baez R, Rojas E, Arias C (2009). Oxidative stress promotes JNK-dependent amyloidogenic processing of normally expressed human APP by differential modification of α-, *β*- and *γ*-secretase expression. *Neurochemistry International*.

[167] Le Freche H, Brouillette J, Fernandez-Gomez F (2012). Tau phosphorylation and sevoflurane anesthesia: an association to postoperative cognitive impairment. *Anesthesiology*.

[168] Whittington RA, Virág L, Marcouiller F (2011). Propofol directly increases tau phosphorylation. *PLoS ONE*.

[169] Papon MA, Whittington RA, El-Khoury NB, Planel E (2011). Alzheimer's disease and anesthesia. *Frontiers in Neuroscience*.

[170] Run X, Liang Z, Zhang L, Iqbal K, Grundke-Iqbal I, Gong C (2009). Anesthesia induces phosphorylation of tau. *Journal of Alzheimer's Disease*.

[171] Giunta B, Fernandez F, Nikolic WV (2008). Inflammaging as a prodrome to Alzheimer's disease. *Journal of Neuroinflammation*.

[172] Toescu EC, Verkhratsky A (2007). The importance of being subtle: Small changes in calcium homeostasis control cognitive decline in normal aging. *Aging Cell*.

[173] Goto M (2008). Inflammaging (inflammation + aging): a driving force for human aging based on an evolutionarily antagonistic pleiotropy theory?. *BioScience Trends*.

[174] Yin F, Sancheti H, Cadenas E (2012). Mitochondrial thiols in the regulation of cell death pathways. *Antioxidants and Redox Signaling*.

[175] Sakono M, Zako T (2010). Amyloid oligomers: formation and toxicity of A*β* oligomers. *FEBS Journal*.

[176] Zhao W-Q, de Felice FG, Fernandez S (2008). Amyloid beta oligomers induce impairment of neuronal insulin receptors. *The FASEB Journal*.

[177] Fu W, Ruangkittisakul A, MacTavish D, Shi JY, Ballanyi K, Jhamandas JH (2012). Amyloid *β* (A*β*) peptide directly activates amylin-3 receptor subtype by triggering multiple intracellular signaling pathways. *The Journal of Biological Chemistry*.

[178] Ferreira IL, Bajouco LM, Mota SI, Auberson YP, Oliveira CR, Rego AC (2012). Amyloid beta peptide 1-42 disturbs intracellular calcium homeostasis through activation of GluN2B-containing N-methyl-d-aspartate receptors in cortical cultures. *Cell Calcium*.

[179] Texidó L, Martín-Satué M, Alberdi E, Solsona C, Matute C (2011). Amyloid *β* peptide oligomers directly activate NMDA receptors. *Cell Calcium*.

[180] Li S, Jin M, Koeglsperger T, Shepardson NE, Shankar GM, Selkoe DJ (2011). Soluble A*β* oligomers inhibit long-term potentiation through a mechanism involving excessive activation of extrasynaptic NR2B-containing NMDA receptors. *Journal of Neuroscience*.

[181] Decker H, Jürgensen S, Adrover MF (2010). N-Methyl-d-aspartate receptors are required for synaptic targeting of Alzheimer's toxic amyloid-*β* peptide oligomers. *Journal of Neurochemistry*.

[182] Malinow R (2012). New developments on the role of NMDA receptors in Alzheimer's disease. *Current Opinion in Neurobiology*.

[183] Dineley KT, Westerman M, Bui D, Bell K, Ashe KH, Sweatt JD (2001). *β*-Amyloid activates the mitogen-activated protein kinase cascade via hippocampal α7 nicotinic acetylcholine receptors: *In Vitro* and *in Vivo* mechanisms related to Alzheimer's disease. *Journal of Neuroscience*.

[184] Buckingham SD, Jones AK, Brown LA, Sattelle DB (2009). Nicotinic acetylcholine receptor signalling: roles in alzheimer's disease and amyloid neuroprotection. *Pharmacological Reviews*.

[185] Inestrosa NC, Toledo EM (2008). The role of Wnt signaling in neuronal dysfunction in Alzheimer's Disease. *Molecular Neurodegeneration*.

[186] Magdesian MH, Carvalho MMVF, Mendes FA (2008). Amyloid-*β* binds to the extracellular cysteine-rich domain of frizzled and inhibits Wnt/*β*-catenin signaling. *Journal of Biological Chemistry*.

[187] O'Neill C, Kiely AP, Coakley MF, Manning S, Long-Smith CM (2012). Insulin and IGF-1 signalling: longevity, protein homoeostasis and Alzheimer's disease. *Biochemical Society Transactions*.

[188] Origlia N, Righi M, Capsoni S (2008). Receptor for advanced glycation end product-dependent activation of p38 mitogen-activated protein kinase contributes to amyloid-*β*-mediated cortical synaptic dysfunction. *The Journal of Neuroscience*.

[189] Yankner BA, Caceres A, Duffy LK (1990). Nerve growth factor potentiates the neurotoxicity of *β* amyloid. *Proceedings of the National Academy of Sciences of the United States of America*.

[190] Yu JT, Wang N, Ma T, Jiang H, Guan J, Tan L (2011). Roles of *β*-adrenergic receptors in Alzheimer’s disease: implications for novel therapeutics. *Brain Research Bulletin*.

[191] Wang D, Govindaiah G, Liu R, de Arcangelis V, Cox CL, Xiang YK (2010). Binding of amyloid *β* peptide to *β*2 adrenergic receptor induces PKA-dependent AMPA receptor hyperactivity. *FASEB Journal*.

[192] Mandelkow E (2012). Biochemistry and cell biology of tau protein in neurofibrillary degeneration. *Cold Spring Harbor perspectives in medicine*.

[193] Kar S, Fan J, Smith MJ, Goedert M, Amos LA (2003). Repeat motifs of tau bind to the insides of microtubules in the absence of taxol. *EMBO Journal*.

[194] Fulga TA, Elson-Schwab I, Khurana V (2007). Abnormal bundling and accumulation of F-actin mediates tau-induced neuronal degeneration in vivo. *Nature Cell Biology*.

[195] Strittmatter WJ, Saunders AM, Goedert M (1994). Isoform-specific interactions of apolipoprotein E with microtubule- associated protein tau: implications for Alzheimer disease. *Proceedings of the National Academy of Sciences of the United States of America*.

[196] Fleming LM, Weisgraber KH, Strittmatter WJ, Troncoso JC, Johnson GVW (1996). Differential binding of apolipoprotein E isoforms to tau and other cytoskeletal proteins. *Experimental Neurology*.

[197] Jing Z, Caltagarone J, Bowser R (2009). Altered subcellular distribution of c-Abl in alzheimer's disease. *Journal of Alzheimer's Disease*.

[198] Jenkins SM, Johnson GVW (1998). Tau complexes with phospholipase C-*γ* in situ. *NeuroReport*.

[199] Sun W, Qureshi HY, Cafferty PW (2002). Glycogen synthase kinase-3*β* is complexed with tau protein in brain microtubules. *Journal of Biological Chemistry*.

[200] Padilla R, Maccioni RB, Avila J (1990). Calmodulin binds to a tubulin binding site of the microtubule-associated protein tau. *Molecular and Cellular Biochemistry*.

[201] Lee YC, Wolff J (1984). Calmodulin binds to both microtubule-associated protein 2 and τ proteins. *Journal of Biological Chemistry*.

[202] Sluchanko NN, Seit-Nebi AS, Gusev NB (2009). Phosphorylation of more than one site is required for tight interaction of human tau protein with 14-3-3zeta. *FEBS Letters*.

[203] Sluchanko NN, Seit-Nebi AS, Gusev NB (2009). Effect of phosphorylation on interaction of human tau protein with 14-3-3*ζ*. *Biochemical and Biophysical Research Communications*.

[204] Hashiguchi M, Sobue K, Paudel HK (2000). 14-3-3*ζ* is an effector of tau protein phosphorylation. *Journal of Biological Chemistry*.

[205] Gauthier-Kemper A, Weissmann C, Golovyashkina N (2011). The frontotemporal dementia mutation R406W blocks tau's interaction with the membrane in an annexin A2-dependent manner. *The Journal of Cell Biology*.

[206] Smet C, Sambo A, Wieruszeski J (2004). The peptidyl prolyl cis/trans-isomerase Pin1 recognizes the phospho-Thr212-Pro213 site on Tau. *Biochemistry*.

[207] Lu P-J, Wulf G, Zhou XZ, Davies P, Lu KP (1999). The prolyl isomerase Pin1 restores the function of Alzheimer-associated phosphorylated tau protein. *Nature*.

